# Short term outcomes of carotid surgery: the real-world experience of a single teaching center

**DOI:** 10.1590/1677-5449.202300332

**Published:** 2024-02-05

**Authors:** Tércio Ferreira Oliveira, Carlos Diego Ribeiro Centellas, Marcelo Bellini Dalio, Edwaldo Edner Joviliano

**Affiliations:** 1 Universidade de São Paulo - USP, Faculdade de Medicina de Ribeirão Preto, Ribeirão Preto, SP, Brasil.

**Keywords:** carotid stenosis, carotid endarterectomy, carotid artery stenting

## Abstract

**Background:**

Surgical treatment of symptomatic extracranial carotid stenosis is well established for preventing neurological events and should adhere to optimal quality standards. However, there is growing concern as to whether results of controlled trials are replicable in real-world settings.

**Objectives:**

To assess a symptomatic carotid stenosis population that underwent surgery and its short-term outcomes in a real-world context at a professional training center.

**Methods:**

Observational study using data collected from medical records from January 2012 to January 2023. Patients undergoing operations for other carotid diseases and with concomitant heart surgery were excluded.

**Results:**

A total of 70 patients undergoing angioplasty or carotid endarterectomy were included. Population subsets undergoing angioplasty or endarterectomy were similar. Differences in anesthetic modality and a longer operative time in the carotid endarterectomy subgroup were statistically significant. There were 4 cases of stroke, only 3 of which (2 minor and 1 major) were related to the index lesion. Thus, the rate of major operation-related stroke was 1.43% and the rate of any lesion-related stroke was 4.29%. There was 1 case of AMI in the angioplasty group and there were no deaths in the sample. The overall rate of major adverse cardiovascular events was 5.71%. There were no statistical differences between the endarterectomy and angioplasty groups regarding the main outcomes.

**Conclusions:**

The rates of outcomes of ischemic stroke, acute myocardial infarction, death, and major adverse cardiovascular events at this center are in line with the rates reported by randomized controlled trials, demonstrating the feasibility of carotid surgery in centers with teaching programs.

## INTRODUCTION

As the second greatest cause of mortality worldwide, after ischemic cardiac disease, the global impact of stroke is well-established.^1,2^

Thromboembolism of the internal carotid artery or middle cerebral artery is one of the principal etiologies of stroke and is responsible for 60% of all disability and 95% of ischemic stroke deaths, accounting for around 23% of total cases.^3,5^

For this reason, and based on the results of three large multicenter studies (NASCET, ECST, and VA309) involving more than 5,000 patients, it was proven that surgical intervention is beneficial for reducing recurrence of cerebral ischemia in symptomatic carotid stenosis exceeding 50%.^5-10^ As a result, the major guidelines recommend surgical intervention as long as the rate of major adverse events is less than 6%.^5,6^

Since studies such as SAPPHIRE and CREST*,* both methods of surgical intervention, open and endovascular, are now considered effective for prevention of new ischemic strokes.^11-13^ While the results of randomized controlled trials are well-established, there is growing concern that they may not be replicable in the context of daily practice.^14-16^ There is increasing appreciation of the value of studies reporting the results achieved in the real world as a means of assessing the effects of interventions tested in controlled clinical trials in uncontrolled populations subject to the effects of all the interference that can affect routine practice.^16^ Studies reporting real-world results, including at centers where health professionals are trained, are needed to evaluate the performance of the interventions that have been proposed. Monitoring the outcomes of institutions’ own units and comparing them with those of other centers also contributes to maintenance of treatment recommendations.

## MATERIALS AND METHODS

An observational study was conducted using data collected from January 2012 to January 2023. A sample size calculation considering a 3% rate of major adverse cardiovascular events (MACE) for carotid endarterectomy (CEA) and a 5% rate for carotid artery stenting (CAS), a 95% confidence interval, and 80% test power estimated 1,500 patients, which is not feasible for a single-center study. Therefore, the study was conducted with a sample of convenience and acknowledgment of its limitations with respect to analytical power and the impact of its results. Data were collected by analysis of electronic medical records from the Hospital das Clínicas, Faculdade de Medicina de Ribeirão Preto, Universidade de São Paulo (HCFMRP-USP). Electronic medical records are archived in the institution’s own system, which was used to retrieve data of importance for the study from patients’ care charts.

The data reported in this study are part of the RHEUNI clinical research project for documentation of vascular disease and it was approved by the Clinical Research Ethics Committee under process number 15695/2011.

### Inclusion and exclusion criteria

**Figure 1:**
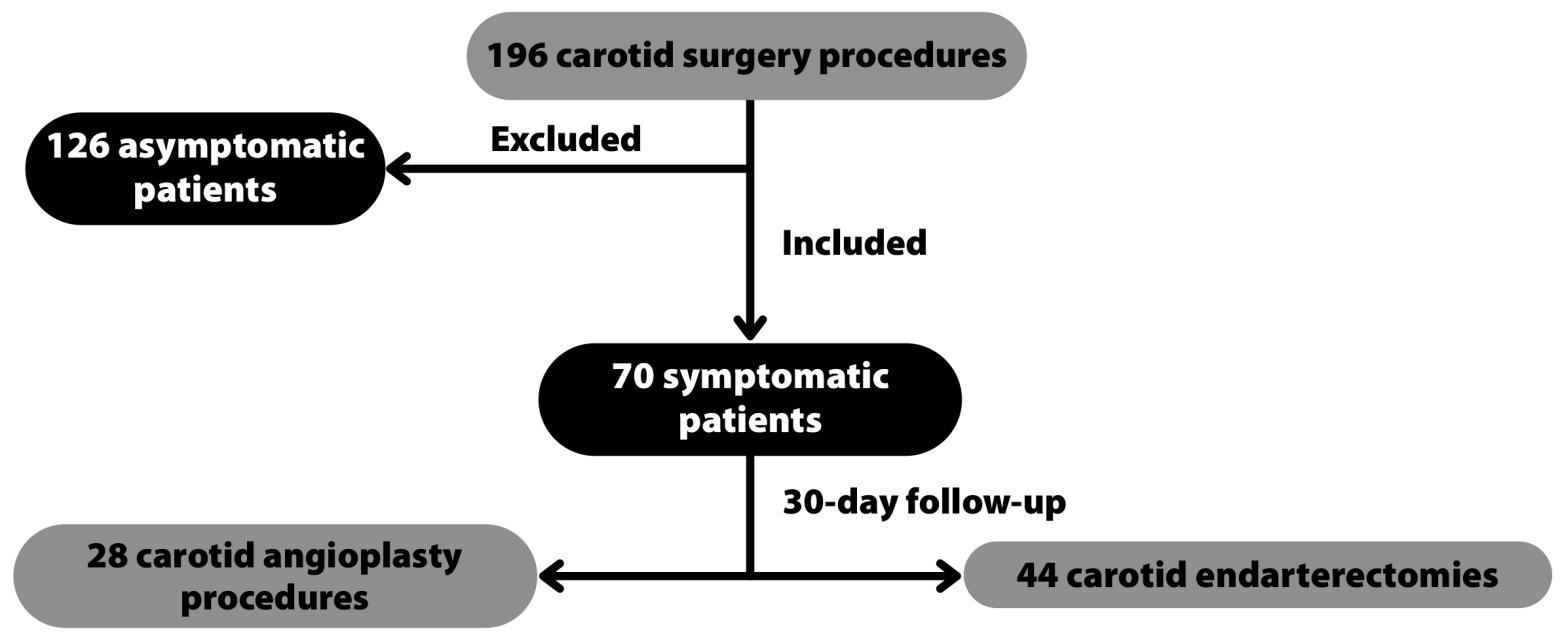
Flow diagram of patient inclusion.

The study included all patients over the age of 18 years who consecutively underwent open or endovascular surgery for symptomatic extracranial carotid stenosis during the period analyzed, as illustrated in Figure 1.

Patients were excluded if they underwent concomitant heart surgery or had carotid dissection, fibromuscular dysplasia, or trauma.

### Definitions

Lesions were considered symptomatic when patients exhibited neurological symptoms attributable to the carotid territory ipsilateral to the stenosing lesion during the previous 180 days, according to the standard Society for Vascular Surgery (SVS) definitions for carotid intervention.^17^ The neurological symptoms attributable to the carotid lesion were those listed in the European Society for Vascular Surgery guidelines: hemispheric sensory impairment (numbness, paresthesia of face, arm, and/or leg); hemispheric motor deficits (weakness of face, arm, and/or leg, or limb coordination impairment); higher cortical dysfunction (dysphasia, aphasia, visual or spatial impairment); amaurosis fugax or transient monocular ischemia; and permanent amaurosis secondary to retinal infarction.^5^

Carotid stenosis exceeding 50% was defined using imaging exams: digital subtraction angiography, applying the NASCET criteria;^8^ Doppler ultrasonography with peak systolic velocity greater than 124 cm/s (end-diastolic velocity greater than 40 cm/s or an internal carotid/common carotid velocity ratio greater than 2.0 was also included as one of the criteria);^18^ or computed tomography or magnetic resonance assessed and reported by the hospital’s radiology team with an imaging report describing stenosis greater than 50%.

### Variables analyzed

Patients’ demographic data and risk factors for carotid stenosis were analyzed. Clinical presentation, degree of stenosis, type of surgery, duration of surgery, type of anesthesia, access complications, and length of hospital stay were all assessed. Additionally, for open surgery, the carotid clamping time, type of patch employed, and use of carotid shunting were also evaluated. In turn, for endovascular surgery, the type of stent employed and use and type of cerebral protection device were also investigated.

The major periprocedural (within 30 days of the procedure) outcomes analyzed were ischemic stroke, all-causes death, acute myocardial infarction (AMI), and combined MACE outcomes (combination of AMI, death, and major stroke).

Ischemic stroke was defined as a de novo focal neurological deficit ipsilateral or contralateral to the lesion with duration exceeding 24 hours and characteristics compatible with focal cerebral ischemia combined with imaging exam confirmation of central nervous system infarct. An AMI was defined as a twofold increase over the reference level of creatine-kinase (CK-MB) or troponin combined with chest pain compatible with ischemia or electrocardiogram with evidence of ischemia. Both these concepts employ definitions presented in the SVS reporting standards.^17^

### Statistical analysis

Statistical analysis was conducted using GraphPad Prism 8.0.1. Descriptive statistics were extracted for general data. The angioplasty and carotid endarterectomy subsets were compared for categorical variables using Fisher’s exact test and, for continuous variables, the Shapiro-Wilk and Kolmogorov-Smirnov tests of normal distribution were applied and then the Mann-Whitney test was employed to compare data with non-normal distribution and Student’s *t* test was used for normally distributed data. A significance level of P < 0.05 was adopted for all tests.

## RESULTS

The study included 70 patients. Table 1 lists their demographic data grouped by type of procedure. No statistical differences in the variables analyzed were detected between the subsets of patients who underwent angioplasty and carotid endarterectomy, as shown in Table 1.

**Table 1:** Demographic data for the study population by type of treatment.

		**CA (N = 26)**		**CEA (N = 44)**		**P (< 0.05)** *		**Total (N = 70)**	
Mean age (SD)		66.9 (±11.8)		67.0 (±7.7)		0.957		67.0(±9.3)	
Female sex		9 (34.6%)		13 (29.5%)		0.790		22 (31.4%)	
Risk factors									
	Arterial hypertension	25 (96.2%)		40 (90.9%)		0.644		65 (92.9%)	
	Diabetes	13 (50.0%)		14 (31.8%)		0.203		27 (38.6%)	
	Dyslipidemia	11 (42.3%)		18 (40.9%)		> 0.99		29 (41.4%)	
	Smoking	18 (69.2%)		27 (61.4%)		0.609		45 (64.3%)	
	Manifest atherosclerotic disease	8 (30.8%)		5 (11.4%)		0.059		13 (18.6%)	
Stenosis grade									
	Severe (> 70%)	19 (73.1%)		40 (90.9%)		0.085		58 (82.9%)	
	Moderate (50-69%)	7 (26.9%)		4 (9.1%)				12 (17.1%)	
Contralateral stenosis		13 (50.0%)		18 (40.9%)		0.619		31 (44.3%)	

SD = standard deviation; CA = carotid angioplasty; CEA = carotid endarterectomy.

* Fisher’s exact test for categorical variables and Student’s *t* test for continuous variables;

The comorbidity with the greatest prevalence was systemic arterial hypertension, present in 92.9% of the patients. Data related to the procedures are shown in Table 2. There was a significant difference in operative time: 77.2 minutes for the angioplasty group and 141.5 minutes for the endarterectomy group, with an overall mean duration of 117.7 minutes. Except for operative time and the anesthetic method employed, there were no other statistical differences in any of the other variables between the angioplasty and endarterectomy subsets.

**Table 2:** Technical characteristics of the procedures.

		**CA (N = 26)**		**CEA (N = 44)**	**P (< 0.05)**	**Total (N = 70)**
General anesthesia		1 (3.84%)		44 (100.0%)	< 0.001*	45 (64.3%)
Use of shunting		-		10 (22.7%)	-	-
Use of patch		-		43 (97.7%)	-	-
Use of protection devices		26 (100.0%)		-	-	-
Mean clamping time (min.)		-		38.10 (±14.4)**	-	-
Mean operative time (min.)		77.2 (±31.9)		141.5 (±31.9)	< 0.001*	117.7 (±45.6)
Mean length of hospital stay (days)		4.0 (±3.02)		3.1 (±1.42)	0.714	3.5 (±2.2)
Access complications		4 (15.4%)		4 (9.09%)	0.457	8 (11.42%)
	Hematoma without reapproach	3 (11.5%)		1 (2.27%)		4 (5.71%)
	Hematoma with reapproach	0 (0.0%)		3 (6.81%)		3 (4.29%)
	Pseudoaneurysm	1 (3.84%)		0 (0.0%)		1 (1.43%)

CA = carotid angioplasty; CEA = carotid endarterectomy.

* Chi-square test for categorical variables and Mann-Whitney (nonparametric) test for continuous variables;

** A total of 47.7% of these data were recorded, with a 95% confidence interval of 33.0-45.7.

All angioplasty procedures were performed with a filter protection device. Open-cell stents were predominantly used, in 53.8% of cases, while 26.9% of the patients had mixed stents and 19.2% had closed-cell stents fitted.

With regard to the endarterectomies, one case was treated using an eversion technique and all of the others were treated using the conventional technique with a patch to reconstruct the arteriotomy, preferably using a bovine pericardium graft, and using a polytetrafluoroethylene (PTFE) graft in one case. The was a 22.7% shunting rate among the endarterectomy procedures. In six cases, the criterion adopted for employing shunting was the retrograde pressure measurement, while in four cases occlusion of the contralateral internal carotid artery had been detected in preoperative tests. The mean duration of carotid clamping observed was 38.1 minutes, but this variable had a high rate of missing data, so the 95% confidence interval was calculated [33.0-45.7].

Table 3 describes the primary outcomes. It is notable that there were no deaths. There were four cases of ischemic stroke during the perioperative period, two of which were minor ischemic strokes, one of which was associated with endarterectomy and one with carotid angioplasty. There was one major ischemic stroke in the carotid angioplasty group. The major ischemic stroke case in the endarterectomy group was in the territory of the posterior circulation and was thus considered to be unrelated to the index lesion. The was just a single case of AMI in the angioplasty group. As such, the rate of major lesion-related stroke was 1.43% (1/70) and the rate of all lesion-related strokes was 4.29% (3/70). The overall MACE rate was 5.71% (4/70). There were no statistical differences between the endarterectomy and angioplasty subsets in terms of the primary outcomes, as shown in Table 3.

**Table 3:** Outcomes according to type of procedure.

				**CA (N = 26)**	**CEA (N = 44)**	**P (< 0.05)** *		**Total (N = 70)**
Ischemic stroke								
	All types			2 (7.69%)	2 (4.55%)	0.624		4 (5.71%)
		Related to index lesion		2 (7.69%)	1 (2.27%)	0.55		3 (4.29%)
			Major ischemic stroke	1 (3.85%)	0 (0.0%)	0.371		1 (1.43%)
			Minor ischemic stroke	1 (3.85%)	1 (2.27%)	> 0.999		2 (2.86%)
		Unrelated to index lesion		0 (0.0%)	1 (2.27%)	> 0.999		1 (1.43%)
			Major ischemic stroke	0 (0.0%)	1 (2.27%)	> 0.999		1 (1.43%)
			Minor ischemic stroke	0 (0.0%)	0 (0.0%)	-		0 (0.0%)
AMI				1 (3.85%)	0 (0.0%)	0.371		1 (1.43%)
Death				0 (0.0%)	0 (0.0%)	-		0 (0.0%)
MACE				3 (11.53%)	1 (2.27%)	0.106		4 (5.71%)

CA= carotid angioplasty; CEA = carotid endarterectomy; AMI = acute myocardial infarction; MACE = major adverse cardiac events.

* Chi-square test for categorical variables.

## DISCUSSION

Although this study was conducted in a different geographic region from the majority of controlled trials conducted to date, the study population had similar baseline characteristics to the samples of the clinical trials of greatest relevance to the topic: NASCET, ECST, CREST, ICSS, EVA3S, and SPACE.^8,9,13,19-21^ Systemic arterial hypertension was the most prevalent comorbidity in the study population. While systemic arterial hypertension is a well-established risk factor for atherosclerotic disease with a relative risk of 1.72 [1.21-2.45],^22^ the rates found in the study population were over 90%, which exceeds the rates in the large studies, which were in the range of 52 to 76%. With the exception of smoking, the prevalence rates of the other comorbidities assessed (diabetes mellitus, dyslipidemia, and manifest atherosclerotic disease) were similar to those found in the literature.

The 64.3% prevalence of active smoking among the patients in the present study, compared with the population means reported in CREST, EVA3S, ICSS, and NASCET (ranging from 23 to 37%), is an indirect indicator of less effective control over comorbidities, which is an integral part of the optimal clinical treatment that should be instituted preoperatively.^8,13,19,20^ In a grouped analysis of four population studies involving screening of asymptomatic populations, smoking conferred a relative risk for carotid stenosis exceeding 50% of 2.3 (1.8-2.8) and a relative risk of 3.0 (2.1-4.4) for stenosis exceeding 70%.^23^ There are also descriptions of the relationship between smoking and atherosclerotic plaque progression and increased intima-media thickness and also with increased risk of late stroke (relative risk increased by 1.9 [1.7-2.2]).^5,24^ These data corroborate the grade 1 recommendation and evidence level A for smoking cessation as a treatment for carotid disease in the most recent European Society for Vascular Surgery guidelines.^5^

Despite the fact that presence of stenosis of the contralateral internal carotid artery may be indicative of greater severity atherosclerotic disease, it is not considered to be a factor related to increased rates of the outcomes stroke/death during the perioperative period.^25,26^ It has highly heterogeneous prevalence in the literature, with rates varying from 2 to 39.6% described in populations. There was contralateral stenosis in 44.3% of the patients in the present study, a rate that exceeds those described for the populations in controlled trials.^19,25-27^

Although the mean duration of carotid endarterectomy surgery (141.5 minutes) was longer than in a large series from the Vascular Quality Initiative database described by Perri et al. (114 minutes), this increased operative time was not correlated with increased MACE rates when compared with the literature or even with other teaching centers.^28,29^ The longer operative time observed in the population of the present study, despite the experience of the surgeons responsible for the procedures, is probably linked to the fact that this service exists in a setting in which new vascular surgeons are trained. A similar explanation can be assumed for the longer carotid clamping time during procedures. The mean clamping time observed in the present study was 38.1 minutes (33.0-45.7), which is a little longer than the 18 to 31 minutes reported by Ferguson et al.^8^ and Malek et al..^30^

### Primary outcomes

Four patients had perioperative ischemic stroke, equating to 5.71% (4/70) of the sample. However, in one of these cases (included according to the study definitions), the ischemic event occurred after discharge and in a different territory to that involved in the surgery and, as such, cannot be considered related to the procedure.

If only events related to the vascular territory of the lesions treated are considered, three ischemic strokes were observed, accounting for 4.29% of the sample. Two of these were minor ischemic strokes and, as such, not incapacitating, and just one case was a major ischemic stroke. Therefore, the rate of incapacitating ischemic stroke associated with the procedure was 1.43%. There was just one case of AMI and none of the patients died during the perioperative period. The total MACE rate was therefore 5.71% (4/70); 11.5% (3/26) for the angioplasty group and 2.27% (1/44) for the endarterectomy group.

Studies that, in common with the present one, report real-world results have found higher rates of adverse events for symptomatic patients than asymptomatic patients (odds ratio [OR] 2.19 [1.58-3.04]).^15,31^ The rate of major adverse events within 30 days can reach 6.9% in some carotid endarterectomy series and can be as high as 8% for angioplasties.^32^

These results confirm that good care practices are being maintained at the institution assessed and corroborate the recommendation to maintain surgical treatment for symptomatic patients at this service, remaining within the acceptable limits (less than 6%) for postoperative complications defined in the literature. The results therefore suggest that carotid surgery remains feasible at teaching centers.

Table 3 lists the results of the subset analysis of patients who underwent angioplasty or carotid endarterectomy, showing there was no statistical difference in primary outcomes. There was a trend to a higher number of MACE in the angioplasty group, which may be related to the lower number of patients in this subset. However, Jalbert et al. assessed real-world outcomes, finding higher rates of adverse events among angioplasty patients.^33^ Notwithstanding, these rates were equivalent to those in endarterectomy patients when the analysis was adjusted for the variable experience of the surgeon, which could be relevant, considering that the center analyzed in the present study is a teaching hospital.

The absence of statistical relevance with relation to the difference in outcomes is in line with the literature, primarily with the three controlled trials that proved that the results of the surgical techniques were equal for symptomatic patients: CREST, EVA-3S, and SPACE.^13,20,21^ In a recent article, Joviliano et al.^28^ reported the prevalence of adverse events associated with endarterectomy and carotid angioplasty performed in real-world settings at five university hospitals. This study found rates of MACE and stroke of 5.92 and 4.61%, respectively, in the angioplasty group and of 4.46% for both outcomes in the endarterectomy group. The present study found similar outcomes, demonstrating that, regardless of being performed within a teaching program, the results achieved with surgical interventions remain within the expected optimum standards.

It should be observed that, despite the comparisons between subsets performed in this study, the data should be evaluated with caution. The objective of this study was to conduct an internal comparison of the study population to validate indications of the different surgical techniques in this population and at this specific center. To enable generalizable comparisons between the surgical methods, the sample size would have had to be around 1,500 patients for a 3% MACE rate after CEA and a 5% rate after CA, with a 95% confidence interval and 80% test power, which is not feasible for a single-center study. Therefore, the relatively low number of patients in the sample attenuates the study’s statistical power and imposes limitations on interpretation and generalization of the data. It is thus advisable that the center be reassessed periodically.

## CONCLUSIONS

The population of patients treated with endarterectomy or angioplasty for symptomatic carotid stenosis at the center investigated had higher prevalence of both systemic arterial hypertension and smoking compared to the populations in the large studies that constitute references in the literature on this subject.

The overall rates of major outcomes (ischemic stroke, AMI, death, and MACE) at this center were similar to those found in randomized clinical trials, which indicates that the service provides good quality care and demonstrates the feasibility of maintaining this type of treatment at teaching centers.
