# Supplementary material for: Short term outcomes of carotid surgery: the real-world experience of a single teaching center
Source: J Vasc Bras. 2024 Feb 5;23:e20230033. doi: 10.1590/1677-5449.202300332 (PMC10903515; doi:10.1590/1677-5449.202300332)
Supplement: Translation

# Translation: Desfechos de curto prazo da cirurgia de carótida: experiência no “mundo real” em um centro único de formação profissional

**DOI:** 10.1590/1677-5449.202300331

**Authors:** Tércio Ferreira Oliveira, Carlos Diego Ribeiro Centellas, Marcelo Bellini Dalio, Edwaldo Edner Joviliano

**Affiliations:** 1 Universidade de São Paulo - USP, Faculdade de Medicina de Ribeirão Preto, Ribeirão Preto, SP, Brasil.

**Keywords:** endarterectomia de carótida, estenose carotídea, angioplastia carotídea

## Abstract

**Contexto:**

A abordagem cirúrgica para estenose carotídea sintomática está consolidada na literatura para a prevenção de eventos neurológicos, devendo seguir padrões ótimos de qualidade. Entretanto, há uma crescente preocupação relacionada à possibilidade ou não de replicar os dados dos trabalhos controlados no mundo real.

**Objetivos:**

Avaliar a população com estenose carotídea sintomática submetida a cirurgia e seus desfechos de curto prazo em um contexto de mundo real em um centro de formação profissional.

**Métodos:**

Tratou-se de um estudo observacional realizado por meio de coleta de dados em prontuário de janeiro de 2012 a janeiro de 2023. Foram excluídos pacientes operados por outras etiologias e com cirurgia cardíaca concomitante.

**Resultados:**

Foram incluídos 70 pacientes submetidos a angioplastia ou endarterectomia carotídea. Os subgrupos populacionais submetidos a angioplastia ou endarterectomia foram semelhantes. Houve diferença estatisticamente relevante quanto à modalidade anestésica e ao tempo cirúrgico maior para o subgrupo de endarterectomia carotídea. Houve quatro casos de acidente vascular encefálico isquêmico, e três deles estavam relacionados à lesão, sendo dois menores e um maior. Dessa forma, a taxa de acidente vascular encefálico maior relacionado à lesão foi de 1,43% e de qualquer acidente vascular encefálico relacionado à lesão, de 4,29%. A taxa total de eventos adversos cardiovasculares maiores foi de 5,71%. Houve um caso de infarto agudo do miocárdio no grupo angioplastia e nenhum óbito. Não houve diferença estatística entre os grupos de endarterectomia e angioplastia quanto aos desfechos principais.

**Conclusões:**

Os desfechos acidente vascular encefálico isquêmico, infarto agudo do miocárdio, óbito e eventos adversos cardiovasculares maiores neste centro são semelhantes aos encontrados em estudos clínicos randomizados, demonstrando viabilidade da manutenção deste tratamento em centros com programas de ensino**.**

## INTRODUÇÃO

Como a segunda maior causa de mortalidade em todo o mundo após a doença isquêmica cardíaca, o impacto global do acidente vascular encefálico (AVE) já está bem estabelecido^1,2^. A repercussão não se limita à mortalidade, mas ao grande dano individual e social, considerando que cerca de metade dos pacientes vítimas dessa doença tornam-se dependentes para a realização de atividades diárias^3-5^.

Figurando entre as principais etiologias do AVE e sendo responsável por 60% do total de incapacidades e por 95% das mortes após AVE isquêmico (AVEi), o tromboembolismo da artéria carótida interna ou cerebral média é responsável por cerca de 23% da totalidade dos casos^3,5^.

Por esse motivo e apoiado em três grandes estudos multicêntricos — NASCET, ECST e VA309 — envolvendo mais de 5.000 pacientes, foi comprovado o benefício da intervenção cirúrgica na redução da recorrência de isquemia cerebral em estenoses carotídeas sintomáticas superiores a 50%^5-10^. Dessa forma, há, nas principais diretrizes, indicação de realização da intervenção cirúrgica, desde que a taxa de eventos adversos maiores seja inferior a 6%^5,6^.

Após estudos como SAPPHIRE e CREST*,* ambas as modalidades de intervenção cirúrgica, aberta e endovascular, foram consideradas eficientes para a prevenção de novos AVEi^11-13^. Apesar dos resultados bem estabelecidos em ensaios randomizados controlados, há crescente preocupação se esses resultados são replicáveis no contexto da prática diária^14-16^. Há uma crescente valorização dos trabalhos envolvendo a descrição de resultados obtidos no mundo real, de forma a avaliar o efeito das intervenções já testadas em ensaios clínicos controlados em uma população não controlada e sob efeito de todas as interferências que podem ocorrer na prática usual^16^. Dessa forma, estudos que demonstrem os resultados do mundo real, incluindo em centros formadores de profissionais, são necessários para avaliação do comportamento das intervenções já propostas. A monitorização dos desfechos das próprias unidades e a comparação com outros centros de referência também contribuem para a manutenção da indicação do tratamento.

## MATERIAIS E MÉTODOS

Foi realizado um estudo do tipo observacional, com coleta de dados de janeiro de 2012 a janeiro de 2023. O cálculo amostral realizado, considerando taxa de eventos adversos cardiovasculares maiores (MACE, de *major adverse cardiovascular events*) de 3% para endarterectomia carotídea (CEA) e de 5% para angioplastia carotídea (CAS) com intervalo de confiança de 95% e uma força de 80%, foi de 1.500 pacientes, o qual seria inviável em um trabalho em centro único. Dessa forma, o presente estudo foi realizado utilizando uma amostra por conveniência e considerando suas limitações quanto ao poder de análise e ao impacto dos resultados. Os dados foram coletados por meio de análise de prontuário eletrônico no Hospital das Clínicas da Faculdade de Medicina de Ribeirão Preto da Universidade de São Paulo (HCFMRP-USP). O prontuário eletrônico está registrado no sistema da própria instituição, e, por meio dele, foram resgatados dados de importância para o trabalho a partir dos registros de assistência ao paciente.

Os dados deste estudo fazem parte do Projeto RHEUNI de pesquisa clínica e registro de doenças vasculares, tendo recebido aprovação do Comitê de Ética em Pesquisa Clínica sob o processo de número 15695/2011.

### Critérios de inclusão e exclusão

Foram incluídos no estudo todos os pacientes com mais de 18 anos que sequencialmente realizaram abordagem cirúrgica aberta ou endovascular por estenose carotídea extracraniana sintomática no período avaliado, conforme descrito na Figura 1.

**Figura 1:** Fluxograma de inclusão dos pacientes.

Foram excluídos pacientes com cirurgia cardíaca concomitante, dissecção carotídea, displasia fibromuscular ou trauma.

### Definições

As lesões consideradas sintomáticas foram aquelas em que os pacientes apresentaram sintomas neurológicos atribuíveis ao território carotídeo ipsilateral à lesão estenosante nos últimos 180 dias, seguindo os padrões de definições para intervenção carotídea da Society for Vascular Surgery (SVS)^17^. Os sintomas neurológicos atribuíveis à lesão considerados são aqueles citados na diretriz da Sociedade Europeia de Cirurgia Vascular: alterações sensitivas hemisféricas (parestesias, hipoestesias da face, braços e/ou pernas); déficits motores hemisféricos (paresia da face, braços e/ou pernas ou alterações da coordenação de membros); disfunções corticais superiores (disfasia, afasia, alterações da visuais ou espaciais); amaurose fugaz ou isquemia monocular transitória; e amaurose definitiva secundária a infarto retiniano^5^.

A estenose carotídea superior a 50% foi definida por meio de exames de imagem: angiografia por subtração digital pela utilização dos critérios do NASCET^8^ ; ultrassonografia Doppler com pico de velocidade sistólica superior a 124 cm/s, sendo que velocidade diastólica final maior que 40 cm/s ou relação de velocidades carótida interna/carótida comum superior a 2,0 foi adicionada como um dos critérios^18^ ; tomografia computadorizada ou ressonância magnética avaliadas e laudadas pela equipe de radiologia do hospital, apresentando laudo com estenose superior a 50%.

### Variáveis avaliadas

Foram analisados os dados demográficos e os fatores de risco para estenose carotídea. A forma de apresentação clínica, o grau de estenose, o tipo de cirurgia realizada, o tempo cirúrgico, o tipo de anestesia, as complicações de acesso e o tempo de internação foram avaliados. Para cirurgias abertas, também foram observados o tempo de clampeamento carotídeo, o tipo de *patch* utilizado e o uso de *shunt* carotídeo. Já no caso de cirurgias endovasculares, foram analisados o tipo de *stent* utilizado e o uso e o tipo de dispositivo de proteção cerebral.

Os principais desfechos avaliados foram AVEi, morte por qualquer causa, infarto agudo do miocárdio (IAM) e desfecho combinado em MACE (combinação entre IAM, morte e AVE maior) ocorridos periprocedimento, ou seja, ao longo de 30 dias após a realização do procedimento.

O AVEi foi definido como novo déficit neurológico focal ipsi ou contralateral à lesão com duração superior a 24 horas e compatível com isquemia cerebral focal associado à confirmação por exame de imagem de infarto do sistema nervoso central. O IAM foi definido como aumento em duas vezes no nível de referência de creatina-cinase (CK-MB) ou troponina associado a dor torácica compatível com isquemia ou eletrocardiograma com evidências de isquemia. Ambos os conceitos seguiram definições apresentadas nos padrões de descrição pela SVS^17^.

### Análise estatística

A análise estatística foi realizada no programa GraphPad Prism 8.0.1. Foi realizada estatística descritiva dos dados gerais. Para comparação entre os grupos de angioplastia e endarterectomia carotídea, foram utilizados o teste exato de Fisher para variáveis categóricas e os testes de Shapiro-Wilk e Kolmogorov-Smirnov para as variáveis contínuas, sendo utilizados para avaliação da normalidade de distribuição dos dados, e, a partir disso, foram utilizados os testes de Mann-Whitney, para amostras com distribuição não normal, e *t* de Student, para amostras com distribuição normal. Para todos os testes, foi adotado um nível de significância de P < 0,05.

## RESULTADOS

Foram incluídos no estudo 70 pacientes, e a Tabela 1 demonstra os dados demográficos agrupados pelo tipo de procedimento realizado. Não foram evidenciadas diferenças estatísticas nas variáveis analisadas quando comparados os grupos de pacientes submetidos a angioplastia ou endarterectomia carotídea, conforme demonstrado na Tabela.

**Tabela 1:** Dados demográficos da população do estudo de acordo com o tipo de tratamento.

		**CAS (N = 26)**	**CEA (N = 44)**	**P (< 0,05)** *		**Total (N = 70)**
Idade média (DP)		66,9 (±11,8)	67,0 (±7,7)	0,957		67,0(±9,3)
Sexo feminino		9 (34,6%)	13 (29,5%)	0,790		22 (31,4%)
Fatores de risco						
	Hipertensão arterial	25 (96,2%)	40 (90,9%)	0,644		65 (92,9%)
	Diabetes	13 (50,0%)	14 (31,8%)	0,203		27 (38,6%)
	Dislipidemia	11 (42,3%)	18 (40,9%)	> 0,99		29 (41,4%)
	Tabagismo	18 (69,2%)	27 (61,4%)	0,609		45 (64,3%)
	Doença aterosclerótica manifesta	8 (30,8%)	5 (11,4%)	0,059		13 (18,6%)
Grau de estenose						
	Grave (> 70%)	19 (73,1%)	40 (90,9%)	0,085		58 (82,9%)
	Moderada (50-69%)	7 (26,9%)	4 (9,1%)			12 (17,1%)
Estenose contralateral		13 (50,0%)	18 (40,9%)	0,619		31 (44,3%)

DP = desvio-padrão; CAS = angioplastia carotídea; CEA = endarterectomia carotídea.

* Teste exato de Fisher para variáveis categóricas e teste *t* de Student para variáveis contínuas;

A hipertensão arterial sistêmica, a comorbidade com maior prevalência, estava presente em 92,9% dos pacientes. Os dados relacionados aos procedimentos estão descritos na Tabela 2. Houve significativa diferença em relação ao tempo cirúrgico: 77,2 minutos para o grupo de angioplastia e 141,5 minutos para grupo de endarterectomia, com média global de 117,7 minutos. Excetuando-se o tempo cirúrgico e a modalidade anestésica, não houve diferença estatística nas demais variáveis avaliadas em relação aos grupos de angioplastia e endarterectomia.

**Tabela 2:** Características técnicas dos procedimentos.

		**CAS (N = 26)**		**CEA (N = 44)**	**P (< 0,05)**	**Total (N = 70)**
Anestesia geral		1 (3,84%)		44 (100,0%)	< 0,001*	45 (64,3%)
Uso de *shunt*		-		10 (22,7%)	-	-
Uso de *patch*		-		43 (97,7%)	-	-
Uso de dispositivo de proteção		26 (100,0%)		-	-	-
Tempo de clampeamento médio (min.)		-		38,10 (±14,4)**	-	-
Tempo cirúrgico médio (min.)		77,2 (±31,9)		141,5 (±31,9)	< 0,001*	117,7 (±45,6)
Tempo de internação médio (dias)		4,0 (±3,02)		3,1 (±1,42)	0,714	3,5 (±2,2)
Complicações de acesso		4 (15,4%)		4 (9,09%)	0,457	8 (11,42%)
	Hematoma sem reabordagem	3 (11,5%)		1 (2,27%)		4 (5,71%)
	Hematoma com reabordagem	0 (0,0%)		3 (6,81%)		3 (4,29%)
	Pseudoaneurisma	1 (3,84%)		0 (0,0%)		1 (1,43%)

CAS = angioplastia carotídea; CEA = endarterectomia carotídea.

* Teste do qui-quadrado para variáveis categóricas e teste (não paramétrico) de Mann-Whitney para variáveis contínuas;

** Foram registrados 47,7% dos dados, com intervalo de confiança de 95% (33,0-45,7).

Todos os procedimentos de angioplastia foram realizados com uso de dispositivo de proteção do tipo filtro. A utilização de *stents* de célula aberta foi predominante, ocorrendo em 53,8% dos casos; 26,9% dos pacientes utilizaram *stents* mistos; e 19,2% utilizaram *stents* de célula fechada.

Quanto às endarterectomias, houve um caso realizado pela técnica de eversão e, nos demais, foi realizada a técnica convencional e utilizado *patch* para reconstrução da arteriotomia, com preferência para uso de enxerto de pericárdio bovino e apenas um caso com uso de enxerto de politetrafluoretileno (PTFE). A taxa de utilização de *shunt* durante o procedimento de endarterectomia foi de 22,7%. Em seis casos, o critério utilizado para uso do *shunt* foi a medida da pressão retrógrada, e, em quatro casos, a oclusão da artéria carótida interna contralateral foi evidenciada em exames pré-operatórios. O tempo médio de clampeamento carotídeo encontrado foi de 38,1 minutos, porém, nessa variável, houve grande perda de dados e, por isso, foi calculado o intervalo de confiança de 95% [33,0-45,7].

Os desfechos principais estão descritos na Tabela 3. Destaca-se que não houve nenhum óbito. Houve quatro casos de AVEi no período perioperatório, dos quais dois eram AVEi menores, um caso estava associado a endarterectomia e um caso estava associado a angioplastia carotídea. Houve um AVEi maior no grupo de angioplastia carotídea. O quadro de AVEi maior pertencente ao grupo de endarterectomia se deu no território da circulação posterior e, portanto, foi considerado AVE não relacionado à lesão. Houve apenas um caso de IAM no grupo de angioplastia. Dessa forma, a taxa de AVE maior relacionado à lesão foi de 1,43% (1/70) e a taxa de qualquer AVE relacionado à lesão foi de 4,29% (3/70). A taxa total de MACE foi de 5,71% (4/70). Não houve diferença estatística entre os grupos de endarterectomia e angioplastia quanto aos desfechos principais, conforme descrito na Tabela 3.

**Tabela 3:** Desfechos de acordo com o tipo de procedimento.

				**CAS (N = 26)**	**CEA (N = 44)**	**P (< 0,05)** *		**Total (N = 70)**
AVEi								
	Qualquer			2 (7,69%)	2 (4,55%)	0,624		4 (5,71%)
		Relacionado à lesão		2 (7,69%)	1 (2,27%)	0,55		3 (4,29%)
			AVEi maior	1 (3,85%)	0 (0,0%)	0,371		1 (1,43%)
			AVEi menor	1 (3,85%)	1 (2,27%)	> 0,999		2 (2,86%)
		Não relacionado à lesão		0 (0,0%)	1 (2,27%)	> 0,999		1 (1,43%)
			AVEi maior	0 (0,0%)	1 (2,27%)	> 0,999		1 (1,43%)
			AVEi menor	0 (0,0%)	0 (0,0%)	-		0 (0,0%)
IAM				1 (3,85%)	0 (0,0%)	0,371		1 (1,43%)
Óbito				0 (0,0%)	0 (0,0%)	-		0 (0,0%)
MACE				3 (11,53%)	1 (2,27%)	0,106		4 (5,71%)

CAS= angioplastia carotídea; CEA = endarterectomia carotídea; AVEi = acidente vascular encefálico isquêmico; IAM = infarto agudo do miocárdio; MACE = eventos adversos cardiovasculares maiores.

* Teste do qui-quadrado para variáveis categóricas

## DISCUSSÃO

Apesar de situada em área geograficamente distinta da maioria dos trabalhos controlados realizados, a população do estudo apresentou características de base semelhantes quando comparada a amostras de ensaios clínicos de grande relevância neste tema: NASCET, ECST, CREST, ICSS, EVA3S, SPACE^8,9,13,19-21^. A hipertensão arterial sistêmica foi a comorbidade mais prevalente na população deste trabalho. A despeito de ser fator de risco bem estabelecido para doença aterosclerótica com risco relativo encontrado de 1,72 [1,21-2,45]^22^ , as taxas de hipertensão arterial sistêmica encontradas na população deste trabalho estão acima de 90% e são superiores às amostras dos grandes estudos, que variaram entre 52 e 76%. As demais comorbidades avaliadas (diabetes melito, dislipidemia e doença aterosclerótica manifesta), com exceção de tabagismo, apresentaram prevalências semelhantes às encontradas na literatura.

A prevalência de 64,3% de pacientes com tabagismo ativo em comparação às médias populacionais encontradas no CREST, EVA3S, ICSS e NASCET (que variaram de 23 a 37%) demonstra indiretamente uma menor efetividade do controle de comorbidades como parte integrante do tratamento clínico otimizado que deve ser instituído desde o pré-operatório^8,13,19,20^. Em análise agrupada de quatro trabalhos populacionais de rastreio em uma população assintomática, o risco relativo atribuível ao tabagismo para estenose carotídea superior a 50% foi de 2,3 (1,8-2,8) e superior a 70% foi de 3,0 (2,1-4,4)^23^. Há também descrição da relação do tabagismo com a progressão da placa aterosclerótica e aumento da espessura médio-intimal, bem como com o aumento do risco de AVE tardio (aumento do risco relativo em 1,9 [1,7-2,2])^5,24^. Esses dados corroboram a indicação grau 1 e nível de evidência A para a interrupção do tabagismo como tratamento da doença carotídea na última diretriz da Sociedade Europeia de Cirurgia Vascular^5^.

Apesar de a presença de estenose da artéria carótida interna contralateral poder ser indicativa de doença aterosclerótica com maior gravidade, ela não é considerada como fator relacionado ao aumento dos desfechos de AVE/óbito no período perioperatório^25,26^. Sua prevalência tem grande heterogeneidade na literatura, com populações variando de 2 até 39,6%. Em 44,3% dos pacientes do presente estudo, havia estenose contralateral, número superior ao descrito nas populações dos estudos controlados^19,25-27^.

A despeito de a média de tempo encontrada para endarterectomia carotídea (141,5 minutos) ser superior à já descrita em grande série do banco de dados Vascular Quality Initiative por Perri et al. (114 minutos), o aumento do tempo cirúrgico não se correlacionou com aumento das taxas de MACE quando comparado com a literatura ou até a outros centro formadores de profissionais^28,29^. O maior tempo cirúrgico encontrado na população do trabalho, apesar da experiência dos cirurgiões responsáveis pelos procedimentos, está provavelmente relacionado ao fato de o serviço estar inserido no contexto de ensino de novos cirurgiões vasculares. Uma justificativa semelhante se supõe para o maior tempo de clampeamento carotídeo durante o procedimento. A média encontrada neste trabalho foi de 38,1 minutos (33,0-45,7), pouco superior aos 18 a 31 minutos encontrados por Ferguson et al.^8^ e Malek et al.^30^.

### Desfechos principais

O AVEi perioperatório ocorreu em quatro pacientes, totalizando 5,71% (4/70) dos casos. No entanto, em um dos casos (incluído por fins de definição do trabalho), o evento isquêmico ocorreu após alta em território divergente da abordagem cirúrgica; desta forma, não podendo ser considerado relacionado com a abordagem.

Se considerados os eventos relacionados ao território vascular da lesão, foram identificados três AVEi, perfazendo 4,29%, sendo dois dos casos de AVEi menores e, portanto, não incapacitantes, e um caso apenas de AVEi maior. Desta forma, a taxa de AVEi incapacitante associado ao procedimento foi de 1,43%. Houve apenas um caso de IAM, e nenhum paciente evoluiu para óbito no período perioperatório. Portanto, a taxa total de MACE foi de 5,71% (4/70); 11,5% (3/26) para o grupo de angioplastia, e 2,27% (1/44) para o grupo de endarterectomia.

Trabalhos que, de forma semelhante a este, mostraram resultados do mundo real evidenciam taxas superiores de eventos adversos quando abordados pacientes sintomáticos em comparação a pacientes assintomáticos (*odds ratio* [OR] 2,19 [1,58-3,04])^15,31^. A taxa de eventos adversos maiores em 30 dias pode chegar a 6,9% em algumas séries para endarterectomias carotídeas e até 8% para angioplastias^32^.

Esses resultados confirmam as boas práticas na prestação do serviço na instituição avaliada e corroboram a manutenção da abordagem cirúrgica dos pacientes sintomáticos nesse serviço que se encontram dentro dos limites aceitáveis (inferiores a 6%) de complicações pós-operatórias impostos na literatura. Os resultados sugerem, portanto, a viabilidade da cirurgia de carótida em centros formadores profissionais.

Quanto à análise de subgrupos de pacientes submetidos a angioplastia ou endarterectomia de carótida, como demonstrado na Tabela 3, não houve diferença estatística quanto aos desfechos principais. Houve uma tendência de maior número de MACE no grupo de angioplastia que pode estar relacionada ao número reduzido de pacientes neste subgrupo. Entretanto, Jalbert et al.^33^ , ao avaliar os desfechos do mundo real, encontraram maiores taxas de eventos adversos em pacientes submetidos a angioplastia^33^. Porém, essas taxas se equipararam às da endarterectomia quando a análise foi feita levando em conta a variável experiência do cirurgião, o que pode ter relevância, considerando que o centro avaliado se encontra em um contexto de ensino.

A ausência de relevância estatística em relação aos desfechos está em consonância com a literatura, principalmente com os três ensaios controlados que provaram a igualdade de resultado das técnicas cirúrgicas para pacientes sintomáticos: CREST, EVA-3S e SPACE^13,20,21^. Em um artigo recente, Joviliano et al.^28^ demonstraram a prevalência de eventos adversos associados a endarterectomia e angioplastia carotídea em um estudo que envolveu cinco centros formadores de profissionais em um ambiente do mundo real. As taxas encontradas nesse trabalho de MACE e AVE foram de 5,92 e 4,61%, respectivamente, no grupo de angioplastia e de 4,46% para ambos no grupo de endarterectomia. No presente trabalho, encontramos desfechos semelhantes, demonstrando que, apesar de ser um programa de ensino, os resultados obtidos a partir das intervenções cirúrgicas se mantêm dentro dos padrões ótimos esperados.

Deve-se observar que, apesar das comparações realizadas no trabalho entre os subgrupos, os dados devem ser avaliados de forma cautelosa. O presente estudo objetivou realizar a comparação interna da população estudada para validação da indicação das diferentes técnicas cirúrgicas na população e do centro específico avaliado pelo trabalho. Para comparação generalizável dos métodos cirúrgicos, o cálculo amostral envolveria em torno de 1.500 pacientes para uma taxa de MACE de 3% para CEA e de 5% para CAS, com intervalo de confiança de 95% e força de 80%, o que seria inviável em um trabalho em centro único. Dessa forma, o número reduzido de pacientes incluídos na amostra apresentado no presente trabalho reduz a força da avaliação estatística e traz limitações na interpretação e generalização dos dados. Portanto, é benéfico que o centro seja reavaliado periodicamente.

## CONCLUSÃO

A população de pacientes submetidos a endarterectomia ou angioplastia por estenose carotídea sintomática no centro avaliado apresenta prevalência superior de hipertensão arterial sistêmica e tabagismo em relação aos grandes estudos de referência da literatura nessa área.

Os desfechos principais globais (AVEi, IAM, óbito e MACE) neste centro são semelhantes aos encontrados em estudos clínicos randomizados, o que indica boa qualidade de assistência prestada pelo serviço e demonstra viabilidade da manutenção deste tipo de tratamento em centros com programas de ensino.
